# Modulatory effects of inhibition on persistent activity in a cortical microcircuit model

**DOI:** 10.3389/fncir.2014.00007

**Published:** 2014-01-31

**Authors:** Xanthippi Konstantoudaki, Athanasia Papoutsi, Kleanthi Chalkiadaki, Panayiota Poirazi, Kyriaki Sidiropoulou

**Affiliations:** ^1^Department of Biology, University of CreteHeraklion, Greece; ^2^Institute of Molecular Biology and Biotechnology, Foundation for Research and Technology – HellasHeraklion, Greece

**Keywords:** prefrontal cortex, NMDA, synchronicity, fast-spiking interneurons, connectivity, parvalbumin interneurons

## Abstract

Neocortical network activity is generated through a dynamic balance between excitation, provided by pyramidal neurons, and inhibition, provided by interneurons. Imbalance of the excitation/inhibition ratio has been identified in several neuropsychiatric diseases, such as schizophrenia, autism and epilepsy, which also present with other cognitive deficits and symptoms associated with prefrontal cortical (PFC) dysfunction. We undertook a computational approach to study how changes in the excitation/inhibition balance in a PFC microcircuit model affect the properties of persistent activity, considered the cellular correlate of working memory function in PFC. To this end, we constructed a PFC microcircuit, consisting of pyramidal neuron models and all three different interneuron types: fast-spiking (FS), regular-spiking (RS), and irregular-spiking (IS) interneurons. Persistent activity was induced in the microcircuit model with a stimulus to the proximal apical dendrites of the pyramidal neuron models, and its properties were analyzed, such as the induction profile, the interspike intervals (ISIs) and neuronal synchronicity. Our simulations showed that (a) the induction but not the firing frequency or neuronal synchronicity is modulated by changes in the NMDA-to-AMPA ratio on FS interneuron model, (b) removing or decreasing the FS model input to the pyramidal neuron models greatly limited the biophysical modulation of persistent activity induction, decreased the ISIs and neuronal synchronicity during persistent activity, (c) the induction and firing properties could not be altered by the addition of other inhibitory inputs to the soma (from RS or IS models), and (d) the synchronicity change could be reversed by the addition of other inhibitory inputs to the soma, but beyond the levels of the control network. Thus, generic somatic inhibition acts as a pacemaker of persistent activity and FS specific inhibition modulates the output of the pacemaker.

## Introduction

Neurons in the prefrontal cortex (PFC) have been shown to exhibit activity that often persists past the end of the stimulus, as recorded *in vivo* during the delay period of working memory tasks (Goldman-Rakic, 1995). This persistent activity corresponds to the on-line representation of a memory for a short period of time. Its emergence has been shown to depend on the balance of excitation, provided by glutamatergic neurons, and inhibition, provided by GABAergic interneurons (Goldman-Rakic, 1995; Compte, 2006) as well as on single neuron dynamics (Sidiropoulou et al., 2009; Yoshida and Hasselmo, 2009). Specifically, activation of NMDA glutamate receptors has been shown to have a highly significant role on supporting stable persistent activity, from computational studies (Wang, 1999; Compte et al., 2000), *in vitro* brain slice experiments (McCormick, 2003) and *in vivo* recordings in monkeys (Wang et al., 2013). Relatively few studies, however, have investigated how interneuron structure and physiology contributes to physiological prefrontal cortical (PFC) function (Rao et al., 1999; Wang et al., 2004).

Interneurons exhibit great diversity in their distribution, connectivity, neurochemistry, synaptic connections and electrophysiological properties. Three main classes have been identified based on their electrophysiological characteristics, namely the FS, the regular-spiking (RS) and the irregular-spiking (IS) interneurons (Markram et al., 2004).

FS interneurons exhibit fast, high-frequency and short duration action potentials. Morphologically, they have been identified as chandelier and basket neurons and they express the calcium-binding protein, parvalbumin (PV). They innervate the soma and the proximal dendritic compartments of pyramidal neurons in the PFC (Wang et al., 2002; Zaitsev, 2005). RS interneurons exhibit RS firing pattern, are mostly double bouquet and Martinotti-type cells andexpress the protein calbidin (CB) (Cauli et al., 1997). They have been shown to innervate the distal dendritic compartments of pyramidal neurons in the PFC. IS neurons exhibit IS firing pattern, are primarily bipolar cells and express the protein calretinin (CR). They project to the dendritic compartments of both PV- and CB- positive cells—suggesting that at least some types of CR-positive cells might be disinhibitory—as well as the distal dendritic compartments of pyramidal neurons in the PFC (Cauli et al., 1997).

Interneuron activity has been shown to contribute to cortical dynamics, network oscillations (Bartos et al., 2007), neuronal synchronization of pyramidal neurons (Guidotti et al., 2005), sensory processing (Börgers and Kopell, 2008), memory function (Jensen et al., 2007), goal-directed behavior (Kvitsani et al., 2013) and social behavior (Yizhar et al., 2011). Specific inactivation of PV interneurons was shown to lead to decreased gamma oscillations in the PFC (Sohal et al., 2009). With regards to working memory function and persistent activity, earlier experimental studies have suggested a role for GABA_A_-mediated inhibition in shaping the memory fields in PFC (Rao et al., 1999). Moreover, *in vitro* experiments suggest that GABA_A_ activation prevents the generation of high frequency epileptiform bursts, while GABA_B_ activation contributes to termination of up-and-down states, a physiological phenomenon related to persistent activity (Mann et al., 2009). Finally, computational studies have implicated the activity of PV/FS interneurons in persistent activity induction and of CB/RS interneurons in mediating the resistance to distractors which can prematurely terminate persistent activity (Wang et al., 2004).

However, there are still many unanswered questions with regards to which biophysical or connectivity properties of the different types of interneurons mediate persistent activity induction, firing frequency characteristics and neuronal synchronicity. In an effort to provide some answers to these questions, we extended a recently developed PFC microcircuit model (Papoutsi et al., 2013) to include the three main types of interneurons, i.e., the FS, regular-spiking (RS), and irregular-spiking (IS) interneurons. We used this modeling tool to dissect the role of different interneuron types in persistent activity and to determine whether the connectivity profile or the physiological properties of these interneuron subtypes mediate their roles in persistent activity.

Our simulations showed that (1) the NMDA current onto the FS interneuron can modulate the induction of persistent activity, but not its maintenance properties, (2) reducing FS model input to the pyramidal neuron models did not allow for NMDA or GABA_B_-dependent modulation of persistent activity induction, and significantly increased the firing frequency, ISI variability and neuronal synchronicity during persistent activity, (3) the firing frequency/ISI variability changes could not be altered by the addition of any other type of inhibitory input to the soma (RS or IS-mediated) and (4) the synchronicity change could be reversed, but beyond the levels of the control network by the addition of non-FS inhibitory input to the soma. Overall, our data suggest that somatic inhibition acts as a pacemaker of persistent activity with the FS interneuron modulating the output of the pacemaker.

## Materials and methods

Four different compartmental model cells were built, based on known electrophysiological data: one pyramidal neuron and three different interneurons, an FS model, an RS model and an IS model. They were connected in a network, which comprised 16 pyramidal models and 4 interneuron models (2 FS models, 1 RS and 1 IS model). Connectivity between the model neurons was based on experimental anatomical and electrophysiological data, as described below. All models are implemented in the Neuron simulation environment (Hines and Carnevale, 2001) and simulations were executed on a xeon cluster (8 core xeon processors).

### Pyramidal neuron model

**Figure 1:**
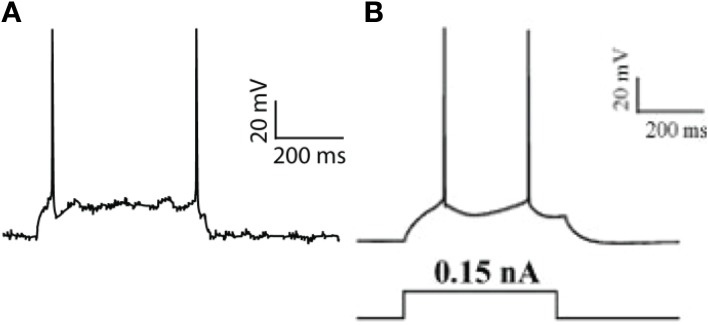
**Pyramidal neuron model validation. (A)** Model response to a current-step pulse at the soma (0.17 nA). **(B)** Experimental response of a PFC layer V pyramidal neuron to a current step-pulse (adapted from Nasif et al., 2004).

The pyramidal neuron model used was based on the one published in Papoutsi et al. (2013) and consists of a soma, a basal, a proximal and a distal dendritic compartment. It includes modeling equations for 14 types of ionic mechanisms, known to be present in these neurons, as well as modeling equations for the regulation of intracellular calcium (same equation as in Papoutsi et al., 2013). The passive and active properties of the pyramidal neuron model was validated according to experimental results of Nasif et al. (2004) (Table 1 and Figure 1). The dimensions of the somatic, axonic, and dendritic compartments of the pyramidal model cell, as well as the passive and active parameters of the model neuron are listed in the supplemental text (Supplemental Tables 1, 2).

**Table 1:** **Input resistance values of the model neurons and those obtained from electrophysiological data**.

	**IR (Model)**	**IR (Experimental)**
Pyramidal	91.3	80 ± 6.8 (Nasif et al., 2004)
FS	250.19	235 ± 68 (Zaitsev, 2005)
RS	487.75	582 ± 195 (Zaitsev, 2005)
IS	545.18	585 ± 137 (Zaitsev, 2005)

### Interneuron models

All three interneuron models included ionic mechanisms for the fast Na^+^, A-type K^+^, and delayed-rectifier K^+^ currents, as well as modeling equations for the regulation of intracellular calcium buffering mechanism (same equations as in Papoutsi et al., 2013). In addition, each different interneuron model subtype included additional ionic mechanisms known to be present in each type (Toledo-Rodriguez et al., 2005), as detailed in the following paragraphs.

### FS interneuron model

**Figure 2:**
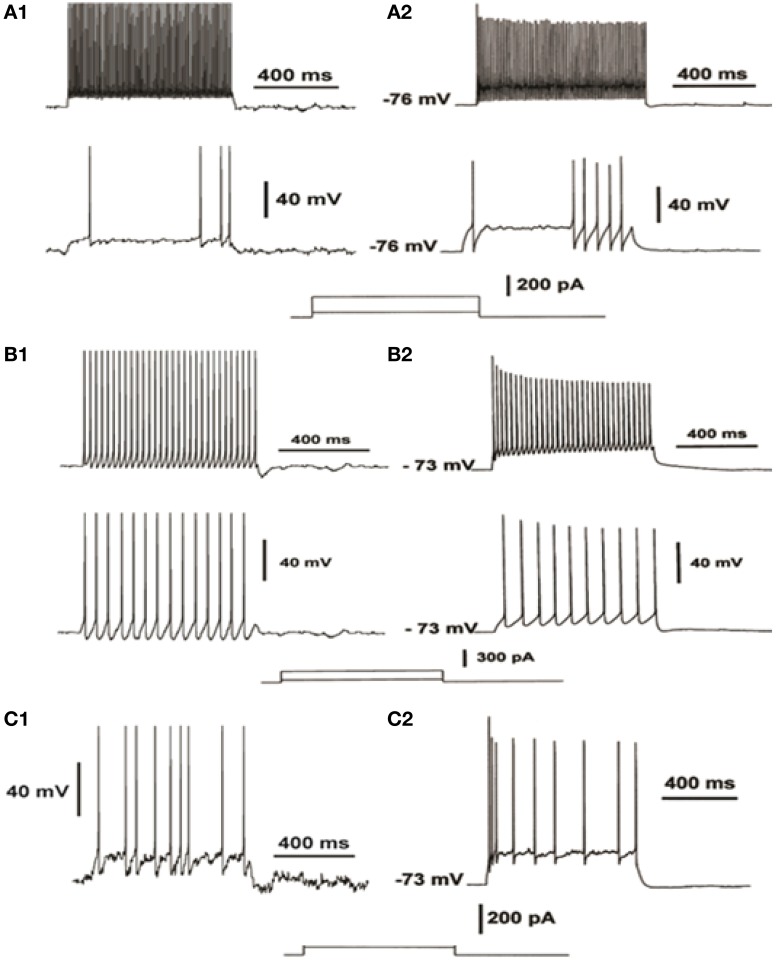
**Validation of the electrophysiological characteristics of the FS interneuron (A), the RS interneuron (B) and the IS interneuron model (C)**. FS neuron model response to increasing depolarizing current injections **(A1)** compared to experimental data from current-clamp recordings (**A2**, adapted from Cauli et al., 1997), reveal the fast-spiking profile of the FS model. RS neuron model response to increasing depolarizing current injections **(B1)** compared to experimental data from current-clamp recordings (**B2**, adapted from Cauli et al., 1997), reveal the regular spiking of the RS model. IS neuron model response to depolarizing current injections **(C1)** compared to experimental data from current-clamp recordings (**C2**, adapted from Cauli et al., 1997), reveal the irregular spiking of the IS model.

The FS interneuron model consisted of three compartments: a somatic, a dendritic and an axonic compartment (Supplemental Table 1). The somatic compartment included mechanisms for the slow K^+^ current (I_Kslow_), the N-type high-threshold activated Ca^++^ current (N-type) and the hyperpolarization-activated cation current (Ih) (Table 2), in addition to the ones mentioned above. The membrane capacitance was set to 1.2 μF/cm2 and axial resistance to 150 ohm/cm (Table 2). The resting membrane potential was adjusted to −73 mV and its resulting input resistance was 250 MΩ (Kawaguchi and Kubota, 1993) (Table 1). The APs of this FS model neuron had short duration and large afterhyperpolarization. It responded to a depolarizing current pulse (0.05 nA, 500 ms) with six spikes, as shown in Figure 2A (top), with an action potential threshold of −53 mV. A depolarizing current of 0.2 nA, 500 ms resulted in a (10 spikes 100 ms) 100 Hz response (Figure 2A, bottom).

**Table 2:** **Active and passive ionic properties of FS interneuron model**.

**FS interneuron mechanisms**	**Soma**	**Axon**	**Dendrite**
Sodium conductance, S/cm^2^	0.135	1.35	0.09
Delayed rectifier K^+^, S/cm^2^	0.036	0.018	0.0075
N-type calcium, S/cm^2^	0.0003	–	–
D-type K^+^, S/cm^2^	0.0000725	–	–
H-current, S/cm^2^	0.00001	–	–
A-type K^+^, S/cm^2^	0.0032	–	0.032
fAHP, S/cm^2^	0.0001	–	–
Calcium diffusion model	Yes	No	No
C_M_ (μF/cm^2^)	1.2	1.2	1.2
R_A_ (ohm/cm)	150	150	150
R_M_ (kΩ cm^2^)	10	10	10

### RS interneuron model

The RS interneuron model consisted of three compartments: a somatic, a dendritic and an axonic compartment (Supplemental Table 1) and included mechanisms for the low-threshold Ca^++^ current (T-type) and the Ih (Table 3). The membrane potential was adjusted to −64 mV (Kawaguchi and Kubota, 1993). The membrane capacitance was set to 1.2 μF/cm2 and the axial resistance to 150 ohm/cm (Table 3). The resulting input resistance is 487 MΩ (Table 1). The model neuron responded to a depolarizing current pulse (0.05, 500 ms) with 15 spikes, with an action potential threshold of −51 mV (Figure 2B, bottom). A depolarizing current of 0.2 nA, 500 ms resulted in a 60 Hz response (Figure 2B, top).

**Table 3:** **Active and passive ionic properties of RS interneuron model**.

**RS interneuron mechanisms**	**Soma**	**Axon**	**Dendrite**
Sodium conductance, S/cm^2^	0.075	0.75	0.018
Delayed rectifier K^+^, S/cm^2^	0.018	0.009	0.009
T-type calcium, S/cm^2^	0.003	–	–
H-current, S/cm^2^	0.000002	–	–
A-type K^+^, S/cm^2^	0.035	–	0.00875
fAHP, S/cm^2^	0	–	–
Calcium diffusion model	Yes	No	No
C_M_ (μF/cm^2^)	1.2	1.2	1.2
R_A_ (ohm/cm)	150	150	150
R_M_ (kΩ cm^2^)	40	40	40

### IS interneuron model

The IS interneuron model consisted of four compartments: a somatic, two dendritic and an axonal compartment, simulating a bipolar cell (Supplemental Table 1), and included mechanisms for slow K+ current, fast Ca^++^-activated K+ current and N-type Ca^++^ current (Table 4). The membrane potential was adjusted to -70 mV (Kawaguchi and Kubota, 1993), the membrane capacitance to 1.2 μF/cm2, and axial resistance to 150 ohm/cm (Table 4). Its input resistance (~545 MΩ) as indicated by electrophysiological data (Zaitsev, 2005) (Table 1). The typical discharge of this cell in response to depolarizing current pulses consisted of the emission of an initial cluster of two to six APs, depending on the level of depolarization, followed by APs emitted at an irregular frequency (Cauli et al., 1997). The discharge frequency increases as a function of the stimulation intensity according to electrophysiological results of (Cauli et al., 1997) (Figure 2C).

**Table 4:** **Active and passive ionic properties of IS interneuron model**.

**IS interneuron mechanisms**	**Soma**	**Axon**	**Dendrites**
Sodium conductance, S/cm^2^	0.015	0.15	0.075
Delayed rectifier K^+^, S/cm^2^	0.018	0.009	0.009
D-type K^+^, S/cm^2^	0.000725	–	–
N-type calcium, S/cm^2^	0.001	–	–
fAHP, S/cm^2^	0.00003	–	–
Calcium diffusion model	Yes	No	No
C_M_ (μF/cm^2^)	1.2	1.2	1.2
R_A_ (ohm/cm)	150	150	150
R_M_ (kΩ cm^2^)	20	20	20

### Microcircuit model

We constructed a microcircuit of 20 neuron models: 16 pyramidal models, based on Papoutsi et al. (2013), 2 FS interneuron models, 1 RS interneuron model and 1 IS interneuron model, so that the relative number of interneurons to pyramidal model neurons was 20% (Dombrowski et al., 2001) and the relative inhibitory input coming from FS interneurons was 50% (Figure 4). Connectivity properties including the location and number of synaptic contacts, the latencies between pairs of neurons, as well as the electrophysiological properties of their synaptic connections, were based on anatomical and electrophysiological data, similar to the values reported in Papoutsi et al. (2013). Specifically, pyramidal neuron models were fully connected recurrently (Wang et al., 2006) at their basal dendrites with latencies drawn from a Gaussian distribution with μ = 1.7 ms and σ = 0.9 (Thomson and Lamy, 2007). Autaptic contacts were also included and were adapted to 1/3 of excitatory connections (Lubke et al., 1996).

### Connectivity

The axon of each pyramidal neuron model projects to the basal dendrite of other pyramidal neuron models. Pyramidal neuron models also projected to the dendrites of FS models, IS model and RS model. However, specificity of synaptic innervations in the neocortex implies that the recurrent network is not randomly arranged (Yoshimura and Callaway, 2005). The axons of the FS interneuron models project to the soma of all pyramidal neuron models. The axon of the RS interneuron model projects to the distal apical dendrite of all pyramidal neuron models (Murayama et al., 2009). The axon of the IS interneuron model projects to the soma of the RS interneuron model, providing disinhibitory input to the micorcircuit, as well as the distal apical dendrite of all pyramidal neuron models. Furthermore, inhibitory autapses are present in the FS interneuron models (Bacci et al., 2003). A summary of synaptic connections present in the microcircuit is described in Table 5.

**Table 5:** **Summary of synaptic connections in the microcircuit**.

**Type of connection**	**Location**	**No. of synapses**	**References**
Thalamocortical (incoming)	Proximal dendrite	120	Kuroda et al., 1998
Pyramidal (recurrent)	Basal dendrite	24	Thomson and Lamy, 2007; Peters et al., 2008
Autapses in Pyr	Basal dendrite	8	Lubke et al., 1996
Pyr -to-FS	Dendrite	12	Markram et al., 2004; Thomson and Lamy, 2007
Pyr -to-RS	Dendrite	14	Markram et al., 2004
Pyr -to-IS	Dendrite	7	Cauli et al., 1997; Markram et al., 2004
Autapses in FS	Soma	1	Bacci et al., 2003
FS -to-Pyr	Soma	15	Tamás et al., 1997a,b; Markram et al., 2004
RS -to-Pyr	Distal dendrite	12	Tamás et al., 1997a,b; Markram et al., 2004
IS -to-Pyr	Distal dendrite	10	Tamás et al., 1997a,b
IS -to-RS	Dendrite	2	Murayama et al., 2009

### Number of synapses

The total number of excitatory synapses to the three types of interneuron models and of inhibitory synapses on the pyramidal neuron model was based on the anatomical data (Tamás et al., 1997a,b; Markram et al., 2004). The total number of inhibitory synapses onto each pyramidal model neuron was 13% of the total excitatory synapses (Peters et al., 2008). A summary of the number of synapses introduced between each type of connection is described in Table 5.

### Validation of the synaptic mechanisms

**Figure 3:**
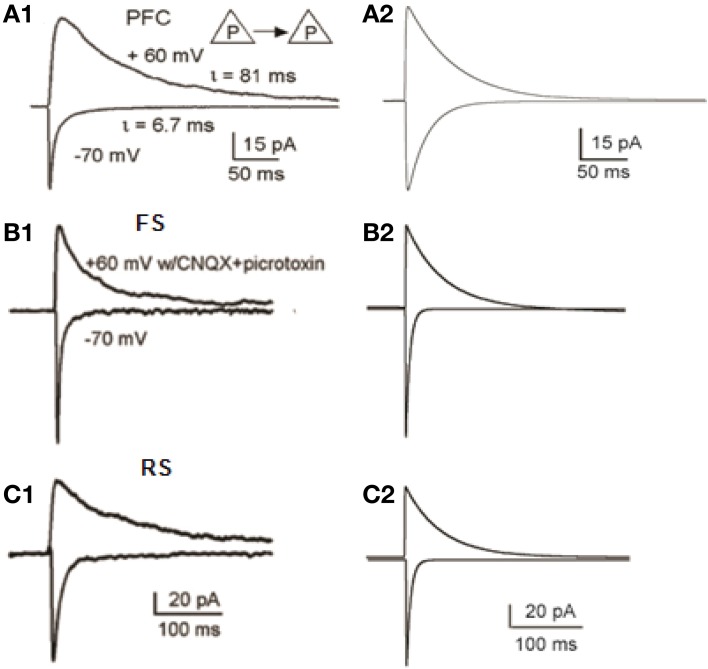
**Validation of Synaptic Properties within the microcircuit. (A1)** Current responses from a layer 5 pyramidal-to-pyramidal pair. When membrane potentials were held at −70 mV, the currents are predominantly mediated by AMPA receptors; whereas at +60 mV, the currents were largely mediated by NMDA receptors (adapted from Wang et al., 2008, Copyright 2008 National Academy of Sciences, USA). **(A2)** Simulated voltage-clamp responses of the pyramidal model neuron at −70 mV (AMPA currents) and at +60 mV (NMDA currents). **(B1,B2)** Experimental **(B1)** and modeling **(B2)** data of AMPA and NMDA currents, in FS interneurons (**B1** is adapted from Wang and Gao, 2009). **(C1,C2)** Experimental **(C1)** and modeling **(C2)** data of AMPA and NMDA currents, in RS interneurons (**C1** is adapted from Wang and Gao, 2009).

The conductances of excitatory and inhibitory synaptic mechanisms were adjusted according to electrophysiological recordings (Thomson and Deuchars, 1997; Angulo et al., 1999; Thomson and Destexhe, 1999; Xiang et al., 2002; Bacci et al., 2003; Woo et al., 2007; Wang et al., 2008; Wang and Gao, 2009). The conductance of a single AMPA-R synapse onto the pyramidal neuron model was adjusted so that it generated a voltage response of 0.1 mV at the soma (Nevian et al., 2007). The NMDA current was validated with a simulated voltage clamp protocol to replicate the results of Wang et al. (2008) (Figure 3A). AMPA- and NMDA- mediated currents were recorded at −70 mV and +60 mV, respectively, in FS and RS neuron models, according to Wang and Gao (2009). Our results correspond to the experimental data, as shown in Figures 3B,C. The relative proportion of NMDA and AMPA receptor mediated synaptic components of the FS models is standardized at 0.5 (Wang and Gao, 2009). The relative proportion of NMDA and AMPA receptor mediated synaptic components of RS models is standardized at 0.8 (Wang and Gao, 2009). In lack of experimental data for the IS neuron model, its AMPA- and NMDA- mediated currents were also simulated to match those of the FS and RS neuron models, whereas the NMDA-to-AMPA ratio was adapted so that the IS interneuron model could fire action potentials during the stimulus.

Furthermore, GABA_A_ receptor mediated currents (IPSCs), between the FS interneuron and the pyramidal neuron were validated, based on Woo et al. (2007) and the GABA_B_ receptor mediated IPSC was validated against experimental data from Thomson et al. (1996) as in Papoutsi et al. (2013). According to Xiang et al. (2002), the amplitude of IPSCs for FS-Pyramidal pairs had a mean value significantly larger than RS-Pyramidal pairs. In particular, the GABA_A_ mediated current between the RS-Pyramidal neuron pair should be 1/10 of the GABA_A_ mediated current between FS-Pyramidal cell pair (Xiang et al., 2002). Due to lack of experimental data for the IPSCs of the IS-Pyramidal neuron pair, GABA_A_ mediated current of this pair was estimated to be 1/10 of the GABA_A_ of RS-Pyramidal pair. The autoinhibition of PV interneurons is much stronger than the inhibition between interneurons of different types, such as IS-RS pairs (Bacci et al., 2003). Autaptic inhibitory currents in FS interneurons evoked a relatively large transient current of 0.35 mA amplitude (Bacci et al., 2003). The aforementioned current was simulated as described in Papoutsi et al. (2013). Across different experiments, the NMDA-to-AMPA ratio of 1.25 and the GABAb-to-GABAa ratio of 0.2 were taken as control state.

### Background noise

In addition, for best simulation of membrane potential fluctuations as observed *in vitro* due to the stochastic ion channel noise (Linaro et al., 2011), an artificial current with Poisson characteristics (mean rate 0.02 Hz) was injected in the soma of all neuron models. Specifically, for the IS neuron model, the amplitude of this mechanism was larger (mean rate 0.035 Hz) (Golomb et al., 2007).

### Stimulation protocol

**Figure 4:**
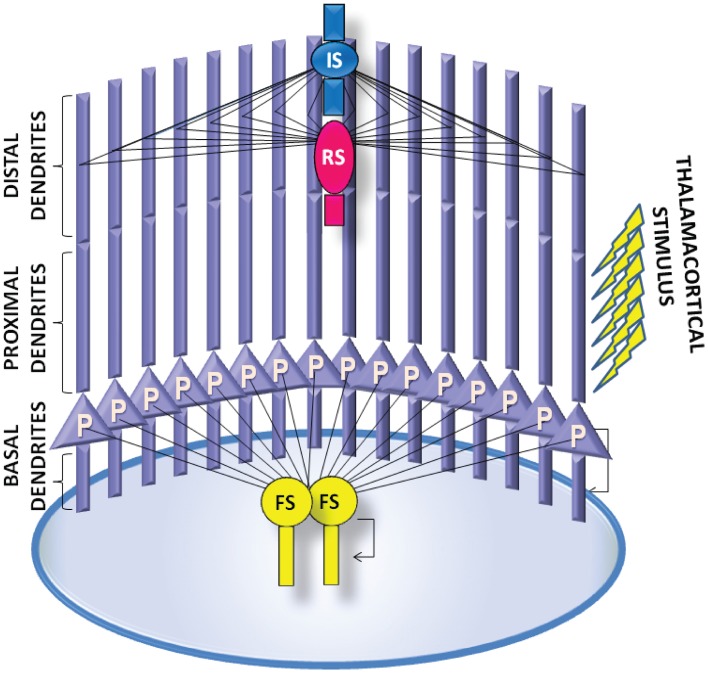
**A schematic of the PFC microcircuit**. The PFC microcircuit consists of 16 pyramidal neuron models (P) and 4 interneuron models; 2 fast-spiking interneuron models (FS), 1 regular spiking interneuron model (RS) and 1 irregular-spiking interneuron model (IS). All neurons are fully connected through recurrent connections. The axon of each pyramidal neuron model projects to the basal dendrite of the other pyramidal neuron models. The axon of the FS interneuron model projects to the soma of all pyramidal neurons models. The axons of the RS and the IS interneuron models project to distal apical dendrite of all pyramidal neuron models. The axon of the IS interneuron model projects to the dendrite of the RS interneuron model. The pyramidal neuron and the FS interneuron models also form autaptic synapses. Persistent activity in the microcircuit is induced by providing external synaptic simulation to all 16 pyramidal neurons in their proximal apical dendrites.

The proximal apical dendrites of the pyramidal neuron models were stimulated with 120 excitatory synapses (containing both AMPA and NMDA receptors), which were activated 10 times at 20 Hz (yellow arrows in Figure 4) (Kuroda et al., 1998). Since neurons within a microcircuit share similar stimulus properties (Yoshimura and Callaway, 2005; Petreanu et al., 2009), the same initial stimulus was delivered to all pyramidal neurons.

### Analysis

Data analysis was performed in Matlab (Mathworks, Inc). Inter-Spike-Intervals (ISIs) were calculated for the neuronal response of each neuron model of the microcircuit during the stimulus and during persistent activity. An average of the ISIs of each neuron of the network, as well as coefficient of variations, in 500 ms time bins was measured for each experimental state.

The Synchronization or de-synchronization of the neurons was measured using the SPIKE-distance measurement, which is sensitive to spike coincidences (Kreuz et al., 2011). For this measurement we obtained the spike trains simultaneously from the neuronal population of the microcircuit and then we calculated the time intervals between successive spikes occurring in any of the participating neurons. If there are no phase lags between the spike trains (neurons fire synchronously) the synchronization index will have values of zero. In general, small values of synchronization index indicate synchronicity, whereas large values indicate asynchronous spiking activity (as in Papoutsi et al., 2013).

As an additional estimation of the synchronization or de-synchronization among spiking neurons in the microcircuit during each different condition, we measured the total number of spikes recorded in 1 ms time bins, and constructed plot with the discrete-time firing rate.

Power spectra were generated on the summed synaptic currents (AMPA, NMDA, and GABA_A_) generated by the pyramidal neurons in the network, averaged for 10 trials, over a 1-s period of steady-state persistent activity, 3 s after the end of the stimulus. The averaged synaptic currents were first decimated and then, the mean square power spectrum was calculated using the periodogram method.

### Model availability

The code of this model in the NEURON simulation environment will be available following contact with the corresponding author and will be posted on ModelDB database upon publication.

## Results

We used a 20-neuron PFC microcircuit model that included 16 biophysically-detailed pyramidal cell models and 4 interneuron models: 2 FS, 1 RS, and 1 IS interneuron model, in order to study the role of these interneuron cell-types in persistent activity emergence and maintenance properties. All modeled neurons were validated against experimental data from intracellular recordings in brain slices (Figure 2—see Methods for details). In addition, the synaptic mechanisms were validated against experimental data (AMPA current, NMDA-to-AMPA ratio, GABA currents) (Figure 3—see Methods for details).

**Figure 5:**
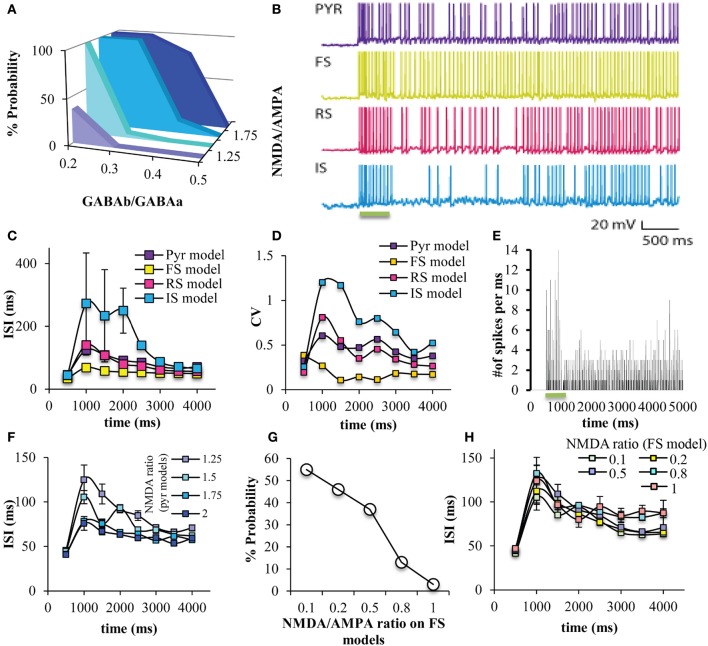
**Persistent activity induction in the network. (A)** The probability of persistent activity induction (measured out of 100 trials) was dependent on the GABAB-to-GABAA and NMDA-to-AMPA ratio on the pyramidal model neurons, as previously seen (Papoutsi et al., 2013). **(B)** Representative traces of all neuron models during the stimulus and during persistent activity. **(C)** Graph showing the ISIs during the stimulus and persistent activity in 500 ms bins, for all neuron models. **(D)** Graph showing the coefficient of variation during the stimulus and persistent activity in 500 ms bins, for all neuron models. The ISI is increased during the initial phase of persistent activity for all model neurons compared to the stimulus. This is not the case for the FS interneuron model. **(E)** Discrete-time firing rate plot showing the number of neurons that fire synchronously during the stimulus and during persistent activity. **(F)** Changing NMDA-to-AMPA ratio on pyramidal neuron models modulated the ISIs, especially during the initial phases of persistent activity. **(G)** Changing NMDA-to-AMPA ratio on FS interneuron models modulated the probability for induction of persistent activity. **(H)** Changing NMDA-to-AMPA ratio on FS interneuron models did not modulated the ISIs.

Persistent activity in the network was induced by an external excitatory stimulus to the apical dendrite (Figure 4). Similar to a smaller version of the microcircuit model (which included 7 pyramidal model neurons and 2 FS interneurons Papoutsi et al., 2013), persistent activity induction was dependent on the GABA_B_-to-GABA_A_ and NMDA-to-AMPA ratio on the pyramidal neuron models (Figure 5A). Each neuron model had a different firing pattern during persistent activity, depending on its own electrophysiological characteristics (Figure 5B). The interspike intervals (ISIs) of the pyramidal neuron model during persistent activity were between 60 and 120 ms, i.e., firing frequency of 8-17 Hz (Figure 5C). The coefficient of variation (CV) of the ISIs, although not very high as observed *in vivo* (Compte, 2006), was greater during persistent activity compared to the CV during the stimulus (Figure 5D). Furthermore, we find that spiking activity of neurons in the network was synchronized both during the stimulus response and the persistent activity, although synchronicity during the stimulus was greater compared to that during persistent activity (Figure 5E). These properties are similar to the corresponding properties observed in persistent activity during working memory tasks (Constantinidis and Procyk, 2004).

Increasing the NMDA-to-AMPA ratio onto pyramidal neuron models decreased the ISIs, especially during the initial phases of persistent activity (Figure 5F), suggesting an increase in the firing frequency. On the other hand, modulating the NMDA-to-AMPA ratio onto FS models does modulate the % probability for induction of persistent activity (Figure 5G) but not the ISIs of the pyramidal neuron model (Figure 5H). This is in accordance with the notion that regulation of NMDA receptors in FS interneurons modulates PFC function (Homayoun and Moghaddam, 2007), although the slow kinetics of the NMDA receptors may not allow for immediate change in network firing (Rotaru et al., 2011). Furthermore, modulating the NMDA-to-AMPA ratio either on pyramidal neuron model or the FS model doesn't have an important effect on the synchronicity among neurons in the microcircuit both during the stimulus and during persistent activity (Supplemental Tables 3, 4). For the rest of the study we used the following conditions: GABA_B_-to-GABA_A_ ratio = 0.2, NMDA-to-AMPA = 1.25 (pyramidal neuron model), and NMDA-to-AMPA = 0.5 (FS interneuron model).

**Figure 6:**
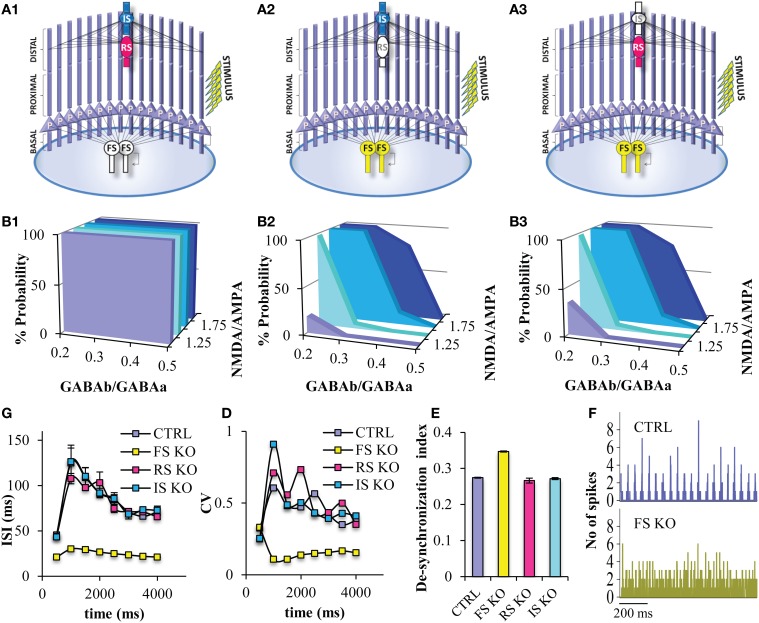
**Persistent activity properties at different simulated “interneuron KO” network models**. **(A)** Graphical representations of the FS interneuron KO network model **(A1)**, the RS interneuron KO network model **(A2)**, the IS interneuron KO network model **(A3)**. **(B)** Graphs showing persistent activity induction across different NMDA-to-AMPA and GABA_B_-to-GABA_A_ ratios in the FS interneuron KO network model **(B1)**, the RS interneuron KO network model **(B2)**, the IS interneuron KO network model **(B3)**. **(C)** Graph showing the ISIs before and during persistent in 500 ms bins for the control and the different “KO” network models. **(D)** Graph showing the CVs of ISIs of the pyramidal neuron model before and during persistent in 500 ms bins for the control and the different “KO” network models. **(E)** Graph showing the de-synchronicity index in the control and different “KO” network models. **(F)** Discrete-time firing rate plot showing the synchronization among all neuron models during persistent activity in the “control” (top) and “FS KO” network models (bottom).

In order to study the role of the different interneuron cell types in persistent activity, we next simulated “knock-out” networks for each interneuron subtype, Thus, we generated a PFC microcircuit without the FS models (“FS KO”) (Figure 6A1), a microcircuit without a RS model (“RS KO” network) (Figure 6A2), and a microcircuit without an IS model (“IS KO” network) (Figure 6A3). We find that the probability for persistent activity induction is always 1, across all GABA_B_-to-GABA_A_ and NMDA-to-AMPA ratios in the “FS KO” network (Figure 6B1), while it is not significantly altered in the “RS KO” and “IS KO” network models (Figures 6B2,3). Therefore, the sensitivity to biophysical modulation is completely lost in the “FS KO” network. In addition, the ISIs during the stimulus and during persistent activity are significantly decreased in the “FS KO” network to 15 ms (i.e., close to 80 Hz frequency), but not significantly altered in the “RS KO” and “IR KO” networks (Figure 6C). As well, the CV of the ISIs of pyramidal neurons during the stimulus and during persistent activity is significantly decreased in the “FS KO” network (Figure 6D). This indicates that the firing rate and its variability of pyramidal neuron models is tightly controlled by the activity of FS interneuron models, but not the RS and IR interneurons. Finally, neuronal synchronicity during persistent activity is also significantly decreased in the “FS KO” network, as evident by the desynchronization index measure and the discrete-time firing rate plot (Figures 6E,F). This is again in accordance with other studies suggesting a contribution of FS interneuron spiking on neuronal synchronization and oscillations (Sohal et al., 2009).

**Figure 7:**
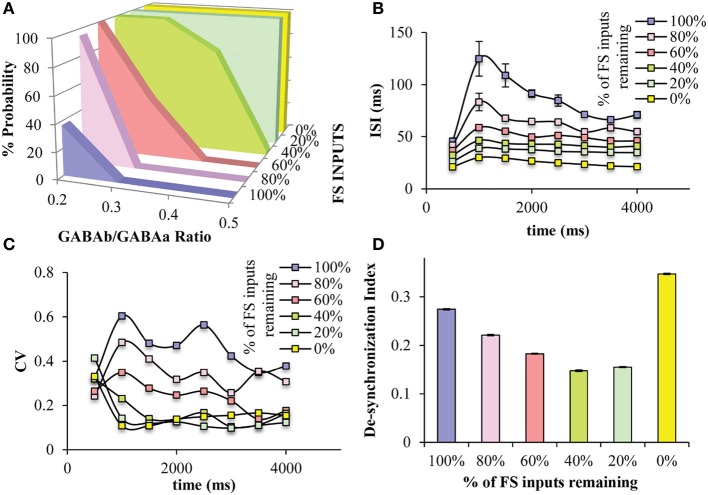
**Effects of decreased number of GABAergic synaptic inputs from the FS models to the pyramidal neuron models. (A)** Decreasing the number of synaptic inputs from the FS model neurons to the pyramidal model neurons increases the range of GABA_B_-to-GABA_A_ ratios, in which persistent activity is induced. When less than 40% of FS inputs are present in the microcircuit, persistent activity is induced 100% across all GABA_B_-to-GABA_A_ ratios. **(B)** As the number of the FS inputs decreases, the ISIs of the pyramidal neuron model decreases. **(C)** As the number of the FS inputs decreases, the CV of the ISIs of the pyramidal neuron model decreases. **(D)** The synchronicity among all neuron models during persistent activity, is significantly reduced when no PV inputs are present in the microcircuit, while it increases by decreasing the number of synaptic inputs from the FS model neurons to the pyramidal model neurons.

Since the “FS KO” net was the only one showing significant differences with regards to persistent activity properties, we wanted to further study the role of the FS interneuron model. Thus, we gradually decreased the number of GABAergic synapses (both GABA_A_ and GABA_B_) from the FS interneuron model onto the pyramidal neuron model in order to simulate a less severe, and possibly more realistic, disruption in the FS neuronal functioning. We find that decreasing the FS model inputs onto the pyramidal neuron model increases the probability for persistent activity induction across the different GABA_B_-to-GABA_A_ (NMDA-to-AMPA = 1.25), while when 40% or less of FS inputs remain, persistent activity is induced across all GABA_B_-to-GABA_A_ ratios tested (Figure 7A). This suggests that once more than 50% of PV inputs are lost, then the PFC microcircuit behaves as if no FS model is present, with regards to induction of persistent activity. This large increase in persistent activity induction renders the microcircuit insensitive to modulation of GABA_B_.

Furthermore, as FS model inputs decrease, the ISIs of the pyramidal neuron model during persistent activity gradually decrease, hence the firing frequency gradually increases (Figure 7B). The variability of ISIs is also decreased when FS inputs decrease to 60% or more, making this index the most sensitive to FS inputs (Figure 7C). Finally, the desyncrhonization index among neuron models in the microcircuit gradually decreases while decreasing the number of FS inputs, but then increases when no FS inputs are present (i.e., “FS KO” net) (Figure 7D). This suggests that synchronicity actually increases when a percentage of FS inputs to the pyramidal neuron models are blocked but then decreases when no inputs are present.

**Figure 8:**
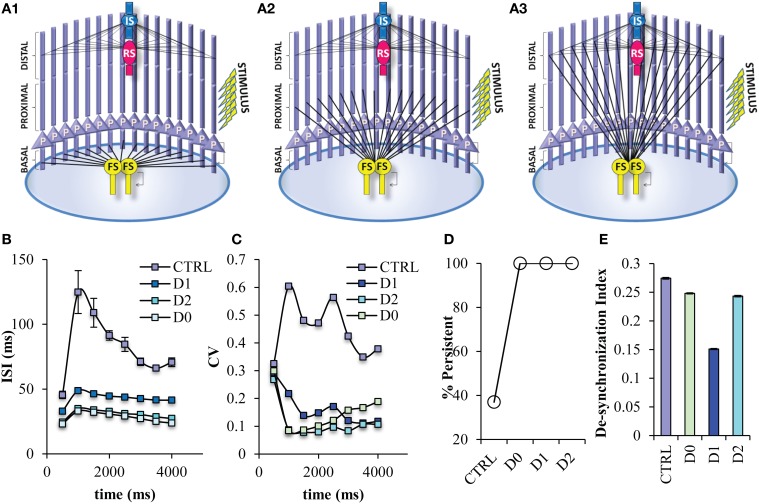
**Persistent activity properties when the FS interneuron model projects to different dendritic compartments**. Different variations of the PFC microcircuit were constructed in order to study the effect of the projection site of FS model neurons onto the pyramidal neurons. **(A)** Graphical representations of the FS interneuron model projecting to the basal (D0) **(A1)**, the proximal (D1) **(A2)**, and distal (D2) **(A3)** dendritic compartment of the pyramidal neuron model. **(B)** Graph showing ISIs before and during persistent in 500 ms bins for the control and the different projecting site networks. The ISIs of pyramidal neuron models are decreased in all microcircuits in which the FS model neurons project to the dendritic compartments of the pyramidal neuron models. **(C)** Graph showing CVs of ISIs before and during persistent in 500 ms bins for the control and the different projecting sites. The CVs of the ISIs of pyramidal neuron models are decreased in all microcircuits in which the FS model neurons project to the dendritic compartments of the pyramidal neuron models. **(D)** Graph showing persistent activity induction in the control and the different FS projecting site networks. **(E)** Graph showing the synchronicity index in the control and different projecting site networks.

Many of the roles of FS neurons on cortical network functions have been attributed to its specific connectivity, specifically the projection of FS neurons to the soma of the pyramidal neurons (Lovett-Barron et al., 2012; Royer et al., 2012). However, by design experimental manipulations cannot differentiate between the target location of an interneuron and its physiological characteristics. So, the next step was to study in detail the role of this specific connectivity by changing the projection site of the FS neuron model to different dendritic locations of the pyramidal neurons other than the soma; on the basal dendrites (D0 net) (Figure 8A1), on the proximal dendrites (D1 net) (Figure 8A2), on the distal dendrites (D2 net) (Figure 8A3). When the FS input is located anywhere else but the soma, then, the probability for induction of persistent activity increases to 100% (Figure 8D), while the ISIs and ISI variability during persistent activity significantly decrease (Figures 8B,C). Furthermore, when the FS model input is located to dendritic locations and not the soma the desynchronization index decreases, suggesting an increase in synchronicity (Figure 8E). Therefore, if FS neuron models do not project to the soma, network activity during persistent activity resembles the state where 50% of less FS inputs to the soma are active (Figure 7).

**Figure 9:**
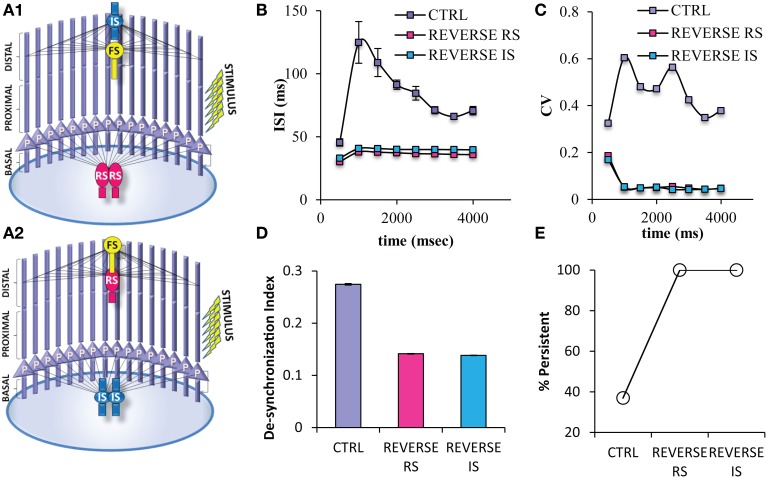
**Different variations of the PFC microcircuit were constructed in order to study the effect of the spiking profile of the neuron model that provides somatic inhibition to the pyramidal neuron**. **(A)** Graphical representations of the microcircuit in which 2 RS interneuron models are projecting to the soma, while 1 FS interneuron model projects to the distal dendritic compartment of pyramidal neuron models (Reverse RS) **(A1)**, and of another microcircuit in which 2 IS interneuron models are projecting to the soma, while 1 FS interneuron model projects to the distal dendritic compartment of pyramidal neuron models (Reverse IS) **(A2)**. **(B)** Graph showing ISIs before and during persistent in 500 ms bins for the control and the two “reverse” states of the network. The ISIs of pyramidal neuron models are decreased in all microcircuits in which the FS model neuron project to the distal dendritic compartments of the pyramidal neuron models while either of the other two neuron models are projecting to the soma of pyramidal neurons. **(C)** Graph showing CVs of ISIs before and during persistent in 500 ms bins for the control and the two “reverse” states of the network. The CVs of ISIs of pyramidal neuron models are decreased in all microcircuits in which the FS model neuron project to the distal dendritic compartments of the pyramidal neuron models while either of the other two neuron models are projecting to the soma of pyramidal neurons. **(D)** Graph showing the synchronicity index in the control and the two “reverse” states of the network. **(E)** Graph showing persistent activity induction in the control and the the two “reverse” states of the network.

Somatic inhibition provided by the FS interneuron seems to be necessary for the induction of proper firing frequencies during persistent activity. In order to eliminate the possibility that the same perisomatic inhibitory effect could be achieved by the other types of interneurons, we modified the network by reversing the projection and number of FS interneuron with RS interneuron (Reverse RS net) (Figure 9A1) and with IS interneuron (Reverse IS net) (Figure 9A2). When one FS interneuron is projecting in distal dendrites of pyramidal neurons, but two RS or two IS interneurons provide somatic inhibition on pyramidal neurons, the probability for induction of persistent activity increases (Figure 9E), while the ISIs and ISI variability during persistent activity significantly decrease (Figures 9B,C). Finally, the desynchronization index decreases in both of the reverse networks (Figure 9D). The above results were resistant to changes in the kinetics of the excitatory synaptic mechanisms of RS and IS models and to changes in the conductance values of the inhibitory synaptic mechanism that the RS and IS models provide to their connected neuron models (Supplemental Figures 1, 2). Our results were also resistant to increasing the frequency (40 Hz) of the stimulation used to initiate persistent activity (Supplemental Figure 3). These results suggest that somatic inhibition provided specifically by the FS neuron is necessary for the firing frequency during persistent activity and allowing for modulation of persistent activity induction. On the other hand, synchronicity of the PFC microcircuit could be increased by changing either the projection of the FS model or changing the physiological profile of the interneuron models projecting to the soma.

**Figure 10:**
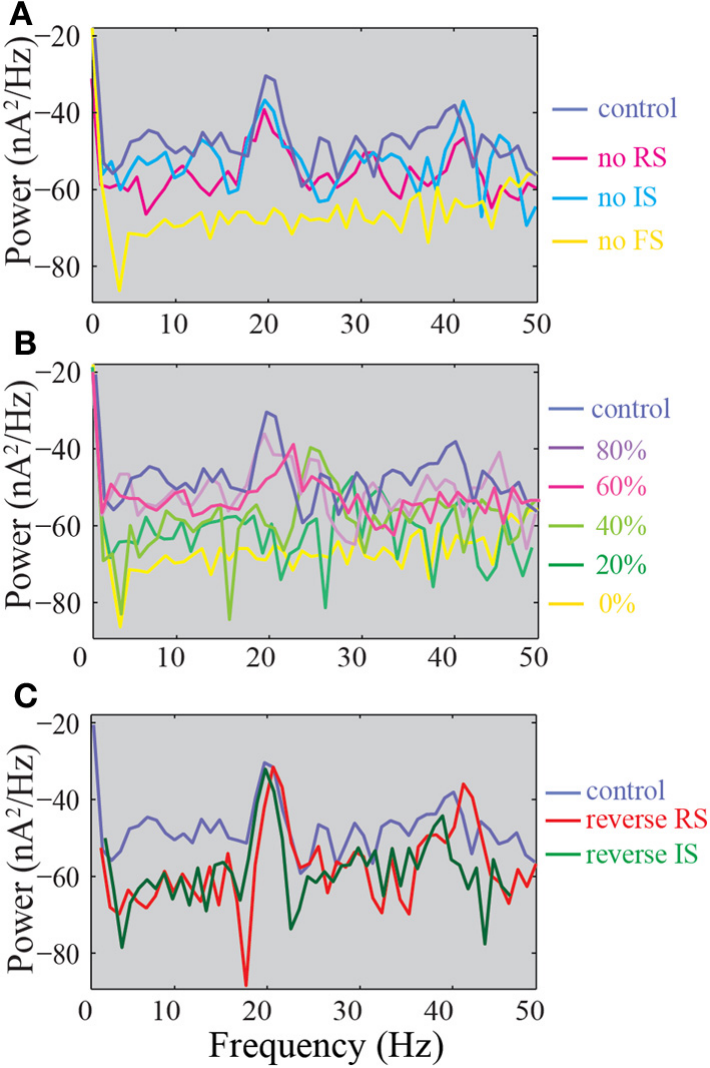
**Power spectra of the summed synaptic activity in the model network. (A)** Power spectra of the control, FS KO, RS KO, and IS KO networks. **(B)** Power spectra of networks with different % of remaining FS inputs on pyramidal models. **(C)** Power spectra of the control compared to reverse RS and reverse IS networks.

In an effort to compare our results to the available literature with regards to changes in network oscillations in the presence of defects in inhibition, we analyzed the power spectra of the summed synaptic currents in the different model networks reported above. In our control network, we observe the presence of a peak in the power spectrum at 20 Hz and a smaller peak at 40 Hz (Figure 10A, dark blue trace). Both peaks are absent in the FS KO network, suggesting a significant role of the FS model neuron in maintaining these oscillations. In addition, only the 40 Hz peak is decreased in the RS KO network, while the power spectrum of the IS KO network is the same as the control (Figure 10A). Both peaks are absent when 50% or less of the FS input to the pyramidal model neuron remain, however, the peak at 40 Hz is already decreased when 80% of the FS inputs remain (Figure 10B). Finally, when the somatic inhibition is provided by the RS or IS neuron, the peaks at 20 and 40 Hz are even larger (Figure 10C). Therefore, our results suggest that the FS input is critical for maintaining network oscillations, and also reveals a novel role of the RS neuron model in maintaining primarily the 40 Hz oscillation.

## Discussion

In this study, we have specifically delineated distinct and specific roles of the FS interneurons in persistent activity properties. First, we identified that NMDA current input onto interneurons only modulates persistent activity induction but not its spiking properties during persistent activity. Second, we find that the FS neuronal inputs to the pyramidal neurons modulate the induction of persistent activity, in an all-or-none way, while the properties of spiking during persistent activity in a gradient manner. Third, moving the FS inputs away from the soma and onto other dendritic compartments has similar effects to completely removing the FS neurons from the network, indicating the significant role of the projecting site of the FS neuron. Finally, we show that replacing somatic inhibition by either the RS or IS neuron models does not reverse the induction or firing frequency changes but does alter neuronal synchronization (increases beyond the control condition).

### NMDA receptors in pyramidal neuron vs. interneurons

The role of pyramidal neuron NMDA currents in persistent activity and working memory tasks is well established (Wang, 2001). Our simulation results reinforce the significance of pyramidal neuron NMDA currents in persistent activity induction and modulation of both induction and the firing properties during persistent activity. However, the role of NMDA receptors on interneurons has received much less attention, particularly from modeling studies of persistent activity. Our modeling results show that an increase in NMDA currents onto FS interneurons decreases the probability for induction of persistent activity, while decreasing the NMDA currents onto FS interneurons increases the probability for induction of persistent activity (Figure 5G). This bidirectional modulation suggests that NMDA receptors at FS interneurons have a critical role in persistent activity induction, and subsequently working memory performance. Our results partly agree with a more generic cortical model (Spencer, 2009), showing the effects of NMDA on FS interneurons on network activity and synchronization. Furthermore, since NMDA on FS interneurons modulates persistent activity induction in our model, we predict that this could impair working memory and other PFC functions. Indeed, removing functional NMDA receptors from FS interneurons has been shown to result in such behavioral defects (Belforte et al., 2009).

### The role of interneurons in neuronal synchronicity and gamma-frequency oscillations

Cortical oscillations, particularly in the gamma-frequency, have been suggested to significantly contribute to several cognitive functions, such as selective attention, perception. These oscillations are thought to reflect synchronous activity of rhythmically firing neurons (Jensen et al., 2007). Activity of PV/FS interneurons has been found in several studies, both experimental and computational, to have a significant role in maintaining the above oscillations and neuronal synchronization (Borgers et al., 2008; Cardin et al., 2009; Sohal et al., 2009; Vierling-Claassen et al., 2010).

Gamma oscillations have been shown to increase the mutual information between incoming synaptic frequency and output of action potentials (Sohal et al., 2009).

In our model, there is a bidirectional modulation of neuronal synchronicity by the FS interneuron. Decreasing the FS input results in increased synchronicity, while a “KO” simulated condition results in decreased synchronicity. Replacing the FS input with either the RS or IR neurons increased synchronicity, but past the levels of the control network. This suggests that the effects of FS input on neuronal synchronicity are complex. Small reductions of FS inputs (20%) result in both increased synchronicity and a small deviation in the firing rate and ISI variability, suggesting that this could be beneficial for the network activity and could possibly result in working memory enhancements. However, 40% or greater reductions in FS-mediated synaptic inputs result not only in increased synchronicity but also in increased firing frequency and decreased ISI variability, indicating a possible defect that could move the network activity toward epileptiform behavior. Therefore, as mentioned in Yu et al. (2004), “it is not the weaker or stronger but an appropriate synchronous state may be of more functional significance in sensory encoding.”

### Changes in interneurons and disease

Converging experimental and clinical evidence suggests that dysfunction in the GABAergic system and the consequent imbalance between excitation and inhibition in the cerebral cortex underlies at least part of the pathophysiology of several neuropsychiatric disorders, such as schizophrenia, epilepsy and autism (Marín, 2012).

In particular, interneuron defects have been associated very strongly with schizophrenia (Lewis et al., 2005). Schizophrenic patients have been shown to express decreased levels of the GABA-synthesizing enzyme, GAD67, and PV (Akbarian, 1995; Volk et al., 2001). In addition, GAD67 and PV are also decreased in several animal models of schizophrenia (Braun et al., 2007; Lodge et al., 2009). Furthermore, both reduced working memory load and reduced power of gamma oscillatory activity in the dorsolateral PFC have been found in schizophrenia (Gonzalez-Burgos and Lewis, 2008), which is also observed when inactivating the PV interneurons with light (Sohal et al., 2009).

Decreased markers of inhibitory transmission, such as number of differentiated PV and CB interneurons is found in animal models of autism (Eagleson et al., 2010; Fu et al., 2012) while a decrease in GAD67 (a marker for inhibitory transmission) is also decreased in human autistic patients (Fatemi et al., 2002). Furthermore, decreased gamma response in the occipital cortex was also found in a human study (Wright et al., 2012).

Epilepsy is another condition that is associated with decreased interneuron populations, as evident mostly from the emergence of epileptic behavior in animal models with reduced number of interneurons (Cobos et al., 2005; Butt et al., 2008; Gant et al., 2009; Peñagarikano et al., 2011). Moreover, epilepsy is characterized by excessive neuronal synchrony (Traub and Wong, 1982), suggesting that decreased interneuron function can also be associated with increased synchrony.

Our results predict that decreases greater than 50% in the number of interneurons or GABAergic synapses lead to disruption in stimulus-specific persistent activity induction. Therefore, any type of stimulus irrespective of neuromodulation could results in persistent activity, a condition that should greatly impair performance in working memory tasks and other PFC-dependent cognitive functions, such as attention and behavioral flexibility.

### Connectivity vs. physiological properties

All three distinct interneuron subtypes differ in their physiological characteristics as well as the location of their synaptic targets. Specifically for the FS interneuron, it has a FS, high frequency physiological profile and it primarily targets the somatic region of the pyramidal neurons (Markram et al., 2004). It has been suggested that the observed function of the specific interneuron subtypes is mostly attributed to the location of their synaptic targets (Wang et al., 2004). The significance of the projection site in the pyramidal neurons has also been shown in our study, since the differences in persistent activity induction, firing frequency and ISI variability are seen when the FS interneuron projects to any dendritic compartment and not the soma. However, our simulations also show that the FS physiological profile is also necessary, since replacing the FS neuron model with either the RS or the IR neuron models at the soma does not reverse the induction, firing frequency and ISI variability changes. Instead, the synchronicity changes are reversed, but not to baseline levels. Specifically, when somatic inhibition is provided by the RS or IS neurons, both the synchronicity and network oscillations are stronger. This suggests that somatic inhibition provided by interneurons with FS activity is absolutely crucial for the different properties of persistent activity. However, any type of inhibition, mediated by either the RS or IR neuron models, can maintain and even further increase neuronal synchronicity. Thus, generic somatic inhibition can serve as a pacemaker of persistent activity, but FS-mediated somatic inhibition is necessary for proper expression of persistent activity properties.

### Model limitations

Our model microcircuit includes different types of model neurons (a pyramidal neuron and 3 different types of interneurons). As seen in the methods, the model network used in this work is heavily constrained with available experimental data. However, sources of inaccuracy can be introduced by the variability of preparations used to produce the experimental data, as well as by the limited availability of data with regards to the specific brain region of our study (prefrontal cortex) and specific layer (layer V). Whenever possible, the data used to validate the models were taken from studies of layer V pyramidal neurons or the different types of interneurons in the prefrontal cortex (Zaitsev, 2005; Peters et al., 2008; Wang et al., 2008; Wang and Gao, 2009). However, in cases there were no available data from the specific region and specific layer, data from non-specified frontal cortex (Kawaguchi and Kubota, 1993; Lubke et al., 1996; Thomson and Lamy, 2007; Woo et al., 2007) or specific primary sensory areas for either pyramidal neurons or interneurons (Cauli et al., 1997; Tamás et al., 1997a,b; Xiang et al., 2002; Toledo-Rodriguez et al., 2005) and were used. Another issue with regards to the available data used to constrain the model is the age of the animals used in the experimental studies. Most of the above studies used to validate our models come from rodents of very young age (up to a month old), although there are some in adult animals, for example (Wang and Gao, 2009). Since it is becoming evident that age plays a very significant role in the cellular physiology and underlying cellular mechanisms (McCutcheon and Marinelli, 2009), our conclusions are limited by the use of the specific available data. Should more specific data from the prefrontal cortex, preferably from adults, become available the specific or future models can be constrained in a more strict way.

Furthermore, there is also variability in the available data with regards to the specific layer examined. Hence, studies using primates suggest that recurrent networks mostly in layer III and to a lesser extent in layer V PFC mediate the persistent activity observed during working memory tasks (Wang et al., 2013). Several models of working memory in the literature (Compte et al., 2000; Wang et al., 2004) simulate layer III recurrent networks (Kritzer and Goldman-Rakic, 1995), in a larger scale compared to the model reported here. The main results, however, with regards to the contribution of NMDA currents, for example, are similar. Even the layer III models constrain their biophysical parameters using layer V electrophysiological recordings (Seamans et al., 1997) or recordings not confined to a specific layer (Connors and Gutnick, 1990; Hammond and Crepel, 1992). In light of recent evidence with regards to differences in biophysical properties of layer III and layer V pyramidal neurons (Bremaud et al., 2007; de Kock, 2013), future modeling studies could be constrained even further with layer specific recordings, possibly revealing layer-specific information coding or persistent activity properties.

### Model predictions and future uses

Our model generates two important predictions that could be tested experimentally. First, it predicts that NMDA current modulation on interneurons only modulates induction of persistent activity and not neuronal excitability or synchronicity during persistent activity. Thus, NMDA receptor blockade specifically in interneurons could increase the emergence of up-and-down states in *in vitro* experiments or increase persistent activity to non-selective stimuli in *in vivo* tasks. Second, it predicts that gradual decrease in the percent of FS-mediated GABAergic synapses will significantly increase the firing rate during persistent activity and decrease the variability of ISIs. For example, in animal models with decreased GABAergic neurons (Peñagarikano et al., 2011; Vidaki et al., 2012), one would expect a very significant increase in the emergence of up-and-down states, during which the neuronal firing rate will be increased and ISI variability decreased.

Furthermore, because of its level of the biophysical detail and extensive validations, the model network can be used as a tool to further delineate the role of interneurons in persistent activity and stimulus-dependent activity. Some examples of studies that could use and/or extend the model include, but are not limited to, (a) identifying the role of the other two interneuron types (RS and IS models) under different stimulation protocols, (b) studying the role of specific biophysical mechanisms on interneurons either on persistent activity or stimulus-dependent activity, and (c) extending the model network to incorporate plasticity rules specific to the different types of interneurons.

### Conflict of interest statement

The authors declare that the research was conducted in the absence of any commercial or financial relationships that could be construed as a potential conflict of interest.
