# Supplementary material for: Modulatory effects of inhibition on persistent activity in a cortical microcircuit model
Source: Front Neural Circuits. 2014 Jan 31;8:7. doi: 10.3389/fncir.2014.00007 (PMC3907788; doi:10.3389/fncir.2014.00007)
Supplement: Supplementary file 1 [file Presentation1.PDF]

Supplemental Figure 1. Properties of persistent activity in response to changes in the synaptic properties of RS neuron models.

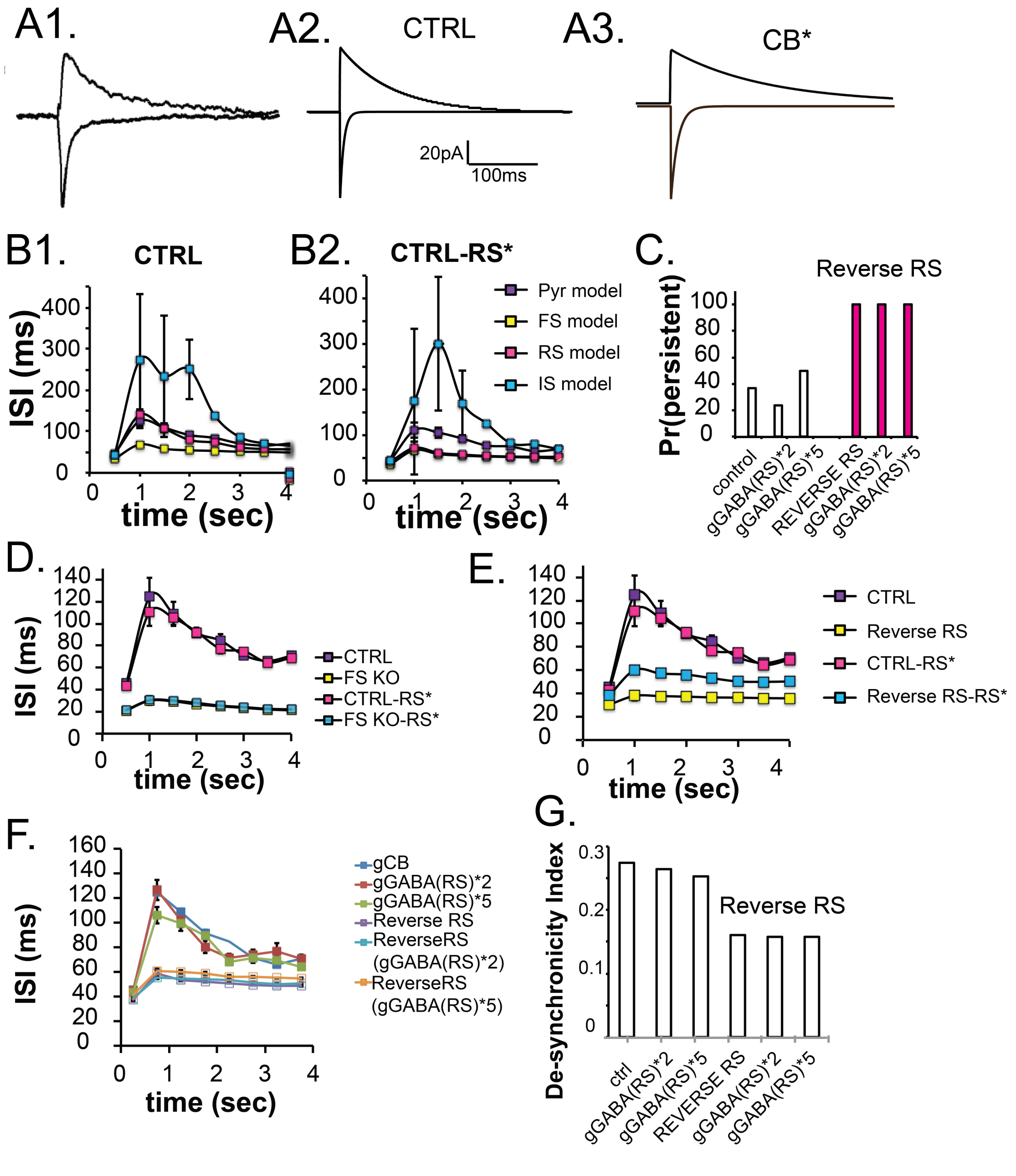

A. Experimental traces, adapted from Wang et al, 2008. A2. Simulated AMPA and NMDA-mediated currents that were used throughout the paper (same as Figure 3) A3. Simulated AMPA and NMDA-mediated currents changes with an increased time constant of decay. These were changing by decreasing the beta parameter in the AMPA (0.1 from 0.18) and the NMDA (0.01 from 0.02) mechanisms. These changes resulted in time constants of decay of 10.5ms for AMPA (compared to 6.5ms) and 67ms for NMDA (compared to 50ms previously) mechanism.

B1. ISIs of all four neuron models as reported in the Figure 4.

B2. ISIs of all four neuron models, when the AMPA and NMDA currents on RS neuron model are changed as mentioned in A.
C. The probability of persistent activity induction is not strongly modulated when the AMPA and NMDA currents on the RS neuron model, either in the control network or the Reverse RS network.
D. ISIs of the pyramidal neuron model in the control and FS KO network, before and after (RS*) the changes in AMPA and NMDA currents on RS neuron models. No changes are reported.
E. ISIs of the pyramidal neuron model in the control and Reverse RS network, before and after (RS*) the changes in AMPA and NMDA currents on RS neuron models. The ISIs of the reverse RS(RS*) network are increased compared to reverseRS, but the qualitatively, there are no differences.
F. ISIs of the pyramidal neuron model in the control and Reverse RS network, when the conductance of the GABA current from the RS neuron model onto the pyramidal neuron model is increased by a factor of 2 or 5. No changes are reported.
G. The de-synchronicity index during the persistent activity does not change in the control or reverse RS network when the conductance of the GABA current from the RS neuron model onto the pyramidal neuron model is increased by a factor of 2 or 5.

Supplemental Figure 2. Properties of persistent activity in response to changes in the synaptic properties of IS neuron models.

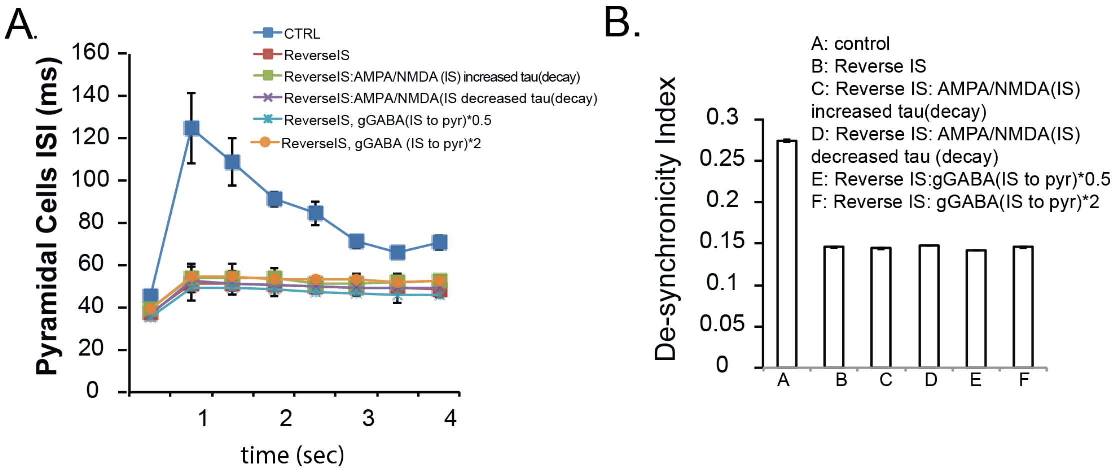

A. ISIs of the pyramidal neuron model in the Reverse IS network, when the conductance of time constants of decay of AMPA and NMDA currents on IS neuron models are either increased or decreased, and when the conductance of the GABA current from the IS neuron model onto the pyramidal neuron model and RS neuron model is halved or doubled. No changes are reported.

G. The de-synchronicity index during the persistent activity does not change in the reverse IS network when the conductance of time constants of decay of AMPA and NMDA currents on IS neuron models are either increased or decreased, and when the conductance of the GABA current from the IS neuron model onto the pyramidal neuron model and the RS neuron model is halved or doubled.

Supplemental Figure 3: Persistent activity properties in response to 40Hz stimulation

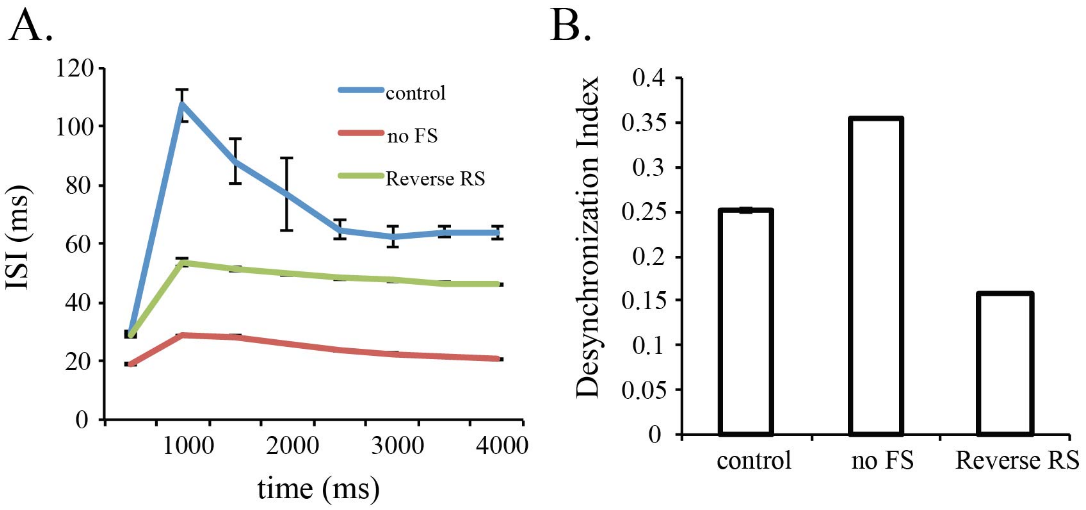

A. ISIs of the pyramidal neuron model in the Reverse IS network, when persistent activity was induced with 40Hz stimulation. Note that the NMDA-to-AMPA ratio was increased to 1.5 in order for the probability for induction of persistent activity to remain the same as our other reported results. No changes are reported.

G. The de-synchronicity index during the persistent activity for the control, the no FS and the ReverseRS networks when persistent activity was induced with 40Hz stimulation.

Supplemental Table 1. Structural parameters of model neurons.

	Length (µm)	Diameter (µm)
**Pyramidal Cell**		
Soma	86.3	10.14
Basal Dendrite	150	1
Proximal Apical Dendrite	400	2.6
Distal Apical Dendrite	400	2.6
Axon	113.22	1.1
**FS Interneuron**		
Soma	27	29
Dendrite	22	7
Axon	115	1.5
**RS Interneuron**		
Soma	42	42
Dendrite	22	7
Axon	113.22	1.1
**IS Interneuron**		
Soma	27	27
Dendrite	22	7
Dendrite	22	7
Axon	113.22	1.1

Supplemental Table 2. Active and passive ionic properties of pyramidal neurons.

Pyramidal Neuron, mechanisms	Soma	Axon	Basal dendrites	Proximal Apical Dendrite	Distal Apical Dendrite
Sodium conductance, S/cm^2	0.108	1.08	0.0108	0.0432	0.0216
Delayed rectifier K$^+$, S/cm^2	5.4e-3	5.4e-3	4.86e-4	2.16e-5	5.4e10-6
Persistent sodium, S/cm^2	18e-7	0	18e-7	54e-7	18e-6
sAHP, S/cm^2	0.025	0	2.5e-5	0.0025	-
A-type K$^+$, S/cm^2	7e-4	0	7e-4	7e-4	7e-4
N-type calcium, S/cm^2	2e-5	0	6e-6	6e-6	0.0001
T-type calcium, S/cm^2	6e-6	0	6e-6	6e-6	6e-7
CaR, S/cm^2	3e-8	0	9e-9	9e-9	15e-8
L-type calcium (a1D)	3e-5	0	3e-5	0	-
L-type calcium (a1C)	1e-5	0	1e-5	1e-5	3e-6
D-type K$^+$, S/cm^2	6e-4	0	0.0006	0.0006	0.0006
fAHP, S/cm^2	2e-4	0	2.2e10-6	2.2e-4	2.2e10-6
H-current, S/cm^2	9e-6	0	9e-6	9e-6	9e-5
Calcium diffusion model	Yes	No	Yes	Yes	Yes
**C_M (μF/cm^2)**	1.2	1.2	2.4	2.4	2.4
**R_A (ohm/cm)**	150	150	150	150	150
**R_M (kΩ cm^2)**	11	11	6	6	6

Supplemental Table 3: Desyncrhonization index at different NMDA-to-AMPA ratios on the pyramidal neuron model

	STIMULATION SPIKE-distance	se	PERSISTENT SPIKE-distance	se
NMDA 1.25	0.117	0.003	0.274	0.001
NMDA 1.5	0.135	0.005	0.253	0.002
NMDA 1.75	0.143	0.004	0.232	0.002
NMDA 2	0.141	0.002	0.227	0.002

Supplemental Table 4: Desyncrhonization index at different NMDA-to-AMPA ratios on the FS interneuron model

	STIMULATION SPIKE-distance	se	PERSISTENT SPIKE-distance	se
NMDAIN 0.1	0.132	0.004	0.253	0.002
NMDAIN 0.2	0.140	0.005	0.261	0.002
NMDAIN 0.5	0.117	0.003	0.274	0.001
NMDAIN 0.8	0.105	0.002	0.288	0.004
NMDAIN 1	0.110	0.002	0.297	0.002

Equations of all biophysical mechanisms used

Almost all equations for the intrinsic and synaptic biophysical mechanisms have been published in Papoutsi et al, 2013, and have not been changed for the current model. Below, we present those equations that have been modified for use in our model.

The NMDA receptor on pyramidal neuron models and interneuron models.

The NMDA receptor (Wang et al., 2008; Wang and Gao, 2009)

$$I = g \cdot (V - E_{rev}) \tag{1}$$

$$g = (R_{on} + R_{off}) \cdot 1(ohm^{-1}) \cdot B \tag{2}$$

$$B = B(V) = \frac{1}{1 + \exp(0.072(mV^{-1}) \cdot (-V)) \cdot (Mg / 3.57(mM))} \tag{3}$$

$$\frac{dR_{on}}{dt} = \frac{synon \cdot R_{\inf} - R_{on}}{R_{\tau}} \tag{4}$$

$$\frac{dR_{off}}{dt} = -\beta \cdot R_{off} \tag{5}$$

$$R_{\inf} = \frac{C_{\max} \cdot \alpha}{C_{\max} \cdot \alpha + \beta} \tag{6}$$

$$R_{\tau} = \frac{1}{C_{\max} \cdot \alpha + \beta} \tag{7}$$

Where, $E_{rev} = 0(mV), Mg = 1(mM), C_{\max} = 1(mM), \alpha = 4(ms^{-1}mM^{-1}), \beta = 0.015(ms^{-1})$

The above β=0.015 (ms^{-1}) is used in the pyramidal neuron model and the IS neuron models. For the FS and RS neuron models, β=0.02 (ms^{-1})

The AMPA receptor

$$I = g \cdot (V - E_{rev}) \tag{8}$$

$$g = (R_{on} + R_{off}) \quad (9)$$

$$\frac{dR_{on}}{dt} = \frac{synon \cdot R_{\inf} - R_{on}}{R_\tau} \quad (10)$$

$$\frac{dR_{off}}{dt} = -\beta \cdot R_{off} \quad (11)$$

$$R_{\inf} = \frac{C_{\max} \cdot \alpha}{C_{\max} \cdot \alpha + \beta} \quad (12)$$

$$R_\tau = \frac{1}{C_{\max} \cdot \alpha + \beta} \quad (13)$$

where $E_{rev} = 0(mV), C_{\max} = 1(mM), \alpha = 10(ms^{-1}), \beta = 0.11(ms^{-1})$

The above β value is used in the pyramidal neuron model. In the FS, RS and IS neuron model, β=0.18 (ms^{-1})

The equation for the fast sodium channel $I_{Na(fast)}$ for the FS neuron model has been previously published in (Sidiropoulou and Poirazi, 2012).

Sidiropoulou K, Poirazi P (2012) Predictive Features of Persistent Activity Emergence in Regular Spiking and Intrinsic Bursting Model Neurons Morrison A, ed. Plos Computational Biology 8:e1002489.

Wang H-X, Gao W-J (2009) Cell Type-Specific Development of NMDA Receptors in the Interneurons of Rat Prefrontal Cortex. Neuropsychopharmacology 34:2028–2040.

Wang H-X, Stradtman GG, Wang X-J, Gao W-J (2008) A specialized NMDA receptor function in layer5 recurrent microcircuitry of the adult ratprefrontal cortex. proceedings of the National Academy of Sciences 105:16791–16796.
